# Supplementary material for: Splice-Junction-Based Mapping of Alternative Isoforms in the Human Proteome
Source: Cell Rep. Author manuscript; Available in PMC 2020 Jan 15. (PMC6961840; doi:10.1016/j.celrep.2019.11.026)
Supplement: 2 [file NIHMS1546469-supplement-2.zip › DF1/PXD010154/Heart/Heart_51_GOT2_ISVAGVTSSNVGYLAHAIHQVTR.pdf]

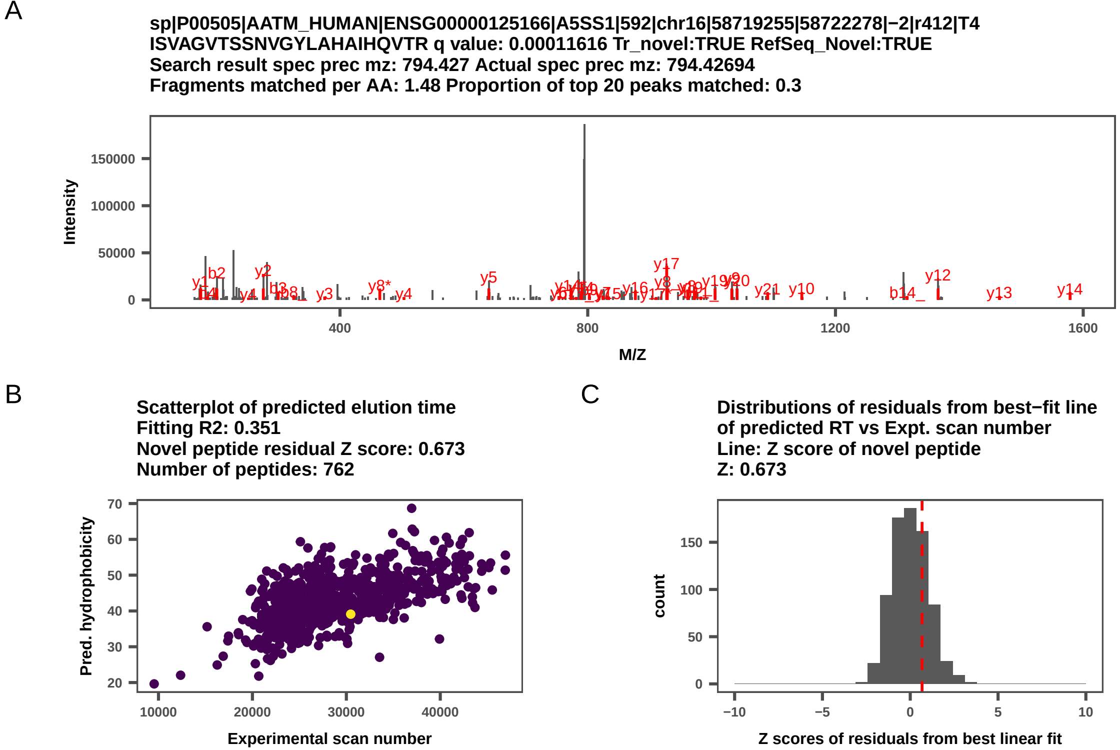
A
sp|P00505|AATM_HUMAN|ENSG00000125166|A5SS1|592|chr16|58719255|58722278|−2|r412|T4
ISVAGVTSSNVGYLAHAIHQVTR q value: 0.00011616 Tr_novel:TRUE RefSeq_Novel:TRUE
Search result spec prec mz: 794.427 Actual spec prec mz: 794.42694
Fragments matched per AA: 1.48 Proportion of top 20 peaks matched: 0.3
Intensity
M/Z
B
Scatterplot of predicted elution time
Fitting R2: 0.351
Novel peptide residual Z score: 0.673
Number of peptides: 762
Pred. hydrophobicity
Experimental scan number
C
Distributions of residuals from best−fit line
of predicted RT vs Expt. scan number
Line: Z score of novel peptide
Z: 0.673
count
Z scores of residuals from best linear fit
